# High-grade serous ovarian carcinoma with mucinous differentiation: report of a rare and unique case suggesting transition from the “SET” feature of high-grade serous carcinoma to the “STEM” feature

**DOI:** 10.1186/s13000-019-0781-9

**Published:** 2019-01-12

**Authors:** Yuichiro Hatano, Maho Tamada, Nami Asano, Yoh Hayasaki, Hiroyuki Tomita, Ken-ichirou Morishige, Akira Hara

**Affiliations:** 1 Department of Tumor Pathology, Gifu University Graduate School of Medicine, Gifu, 501-1194 Japan; 2 Department of Obstetrics and Gynecology, Gifu University Graduate School of Medicine, Gifu, 501-1194 Japan

**Keywords:** Ovary, High-grade serous carcinoma, SET feature, Mucinous differentiation, *TP53*

## Abstract

**Background:**

High-grade serous carcinoma, a representative high-grade ovarian carcinoma, is believed to be closely associated with a *TP53* mutation. Recently, this category of ovarian carcinoma has gained increasing attention owing to the recognition of morphological varieties of *TP53*-mutated high-grade ovarian carcinoma. Herein, we report the case of a patient with high-grade serous carcinoma with mucinous differentiation.

**Case presentation:**

A 59-year-old postmenopausal woman was referred to the gynecologist because of abnormal vaginal bleeding. The radiological assessment revealed an intrapelvic multicystic mass, which was interpreted as an early right ovarian cancer and then removed by radical surgery. Histologically, the cancer cells were found in the bilateral ovaries and para-aortic lymph nodes. The cancer cells showed high-grade nuclear atypia and various morphologies, including the solid, pseudo-endometrioid, transitional cell-like (SET) pattern, and mucin-producing patterns. Benign and/or borderline mucin-producing epithelium, serous tubal intraepithelial carcinoma, and endometriosis-related lesions were not observed. In immunohistochemistry analyses, the cancer cells were diffuse positive for p53; block positive for p16; partial positive for WT1, ER, PgR, CDX2 and PAX8; and negative for p40, p63, GATA3, Napsin A, and vimentin*.* The Ki-67 labeling index of the cancer cells was 60–80*%.* Direct sequencing revealed that the cancer cells contained a missense mutation (c.730G>A) in the *TP53* gene.

**Conclusion:**

Mucinous differentiation in high-grade serous carcinoma is a rare and unique ovarian tumor phenotype and it mimics the phenotypes of mucinous or seromucinous carcinoma. To avoid the misdiagnosis, extensive histological and immunohistochemical analyses should be performed when pathologists encounter high-grade mucin-producing ovarian carcinoma. The present case shows that the unusual histological characteristic of high-grade serous carcinoma, the “SET” feature, could be extended to the solid, transitional, endometrioid and mucinous-like (STEM) feature.

## Background

Traditionally, classification of ovarian tumor is principally based on morphology [1, 2]. The current five major ovarian epithelial cancers consist of high-grade and low-grade serous, mucinous, clear cell, endometrioid carcinoma [3, 4]. Each ovarian carcinoma histological type shows a specific cellular phenotype and gene expression profile, which resembles that of the normal corresponding epithelium [5]. For example, serous carcinoma cells partially look like the fallopian tubal epithelial cells. In other words, the evidence of cellular differentiation warrants the validity of morphology-based tumor classification in ovarian cancer.

However, despite the communality of tumor cell differentiation, high-grade serous carcinoma (HGSC) quite differs from low-grade serous carcinoma (LGSC) in regard to clinicopathological features [6, 7]. HGSC is the most aggressive and common ovarian carcinoma that is believed to be closely associated with a *TP53* mutation (type II carcinoma), whereas LGSC, as well as the other major ovarian cancer histological types, is one of the indolent ovarian malignancies that develops from the benign and/or borderline counterparts through multi-step carcinogenesis (type I carcinoma) [8]. To distinguish these two serous carcinomas, p53 immunohistochemistry is a useful tool, which is a surrogate molecular test for *TP53* mutation [9]. Indeed, HGSC almost always harbors the *TP53* mutation [10], which plays an important role in high-grade ovarian carcinogenesis [11]. Notably, five gynecological pathologists of the United States reassessed the *TP53* mutation-lacking HGSC of the TCGA study [10], and then concluded that molecular alteration of the *TP53* gene is essential for diagnosis of HGSC [12]. In contrast, LGSC rarely harbors the *TP53* mutation [13].

Recently, the concept of *TP53* mutation-based high-grade ovarian tumors has garnered increasing attention. Soslow et al. reported that high-grade ovarian carcinoma with unusual morphologies, including the solid, pseudo-endometrioid and transitional cell carcinoma-like (SET) pattern, additionally exhibits the typical molecular aberration of type II carcinoma [14]. This alternative HGSC SET variant indicates that the molecular analysis of ovarian carcinoma is necessary to current tumor classification.

We herein report the case of a patient with a HGSC with mucinous differentiation. The ovarian tumor of the present case is regarded as a rare carcinoma and, thus, could possibly be misdiagnosed as a mucinous or seromucinous carcinoma owing to the morphological finding. Recognition of unique characteristics of this tumor could further expand the concept of ovarian type II carcinoma and prevent the underestimation of its malignant potential.

## Case presentation

### Clinical history

**Fig. 1:**
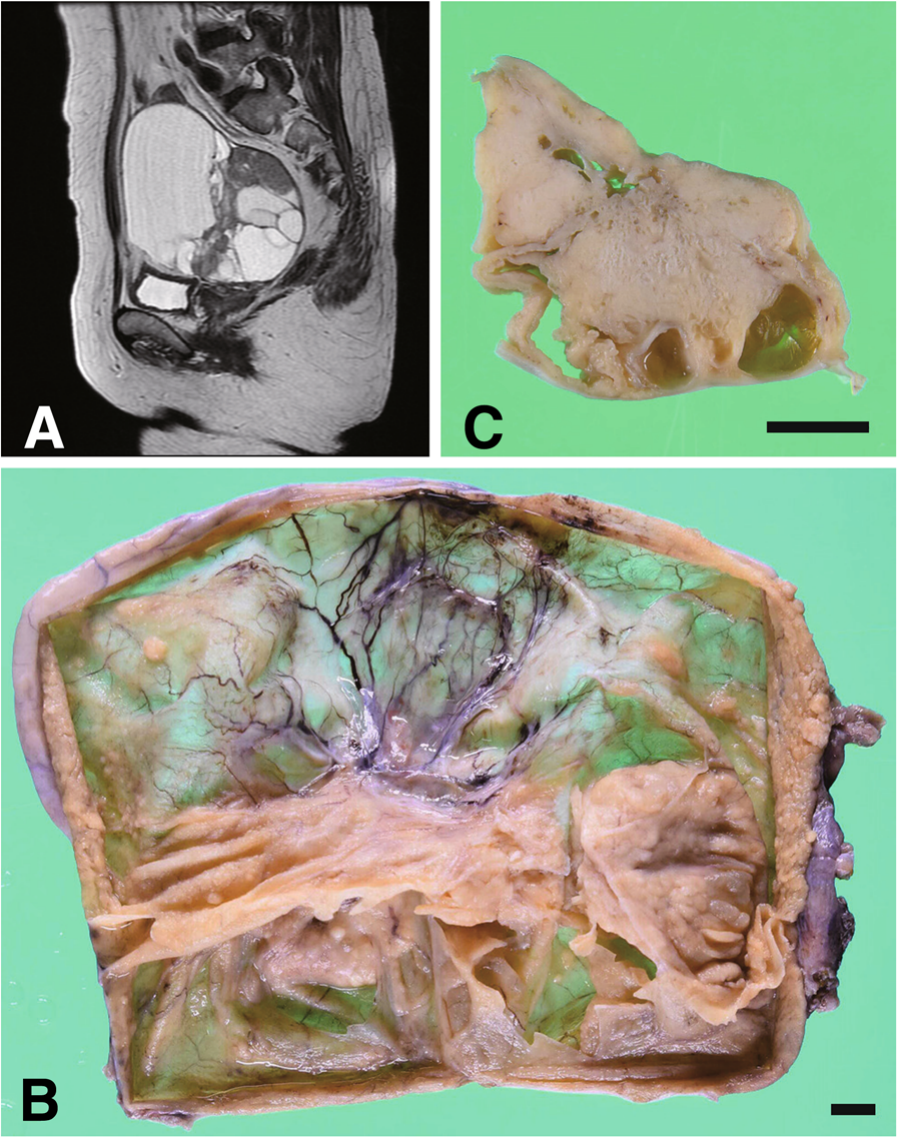
Overview of the ovarian cancer. **a** Representative sagittal view of magnetic resonance imaging. The inside (**b**) and cut surface (**c**) of the ovarian tumor. Black bars: 1 cm

A 59-year-old postmenopausal woman (gravida 2, para 2) was referred to the gynecologist because of abnormal vaginal bleeding. She had a past medical history of hyperthyroidism and was on thyroid hormone replacement therapy at presentation. She denied any familial history of ovarian and/or breast cancer. Blood tests revealed that serum CA125 was slightly high (96.2 U/mL). Pelvic ultrasonography was notable for a polycystic mass, measuring 117 × 71 mm, adjacent to the normal-appearing uterus. Abdominopelvic computed tomography showed a polycystic and solid mass, measuring 135 × 92 × 100 mm, which was connected to the right ovarian vein. In addition, contrast enhanced-magnetic resonance imaging revealed enhancement in the septal area and heterogeneity of intracystic signal intensity, suggesting ovarian mucinous carcinoma (Fig. 1a). Her disease was diagnosed as early ovarian cancer, FIGO Stage IA (cT1aN0M0); then, she received total hysterectomy with bilateral salpingo-oophorectomy, omentectomy, intra-pelvic and para-aortic lymphadenectomy.

### Pathological findings

**Fig. 2:**
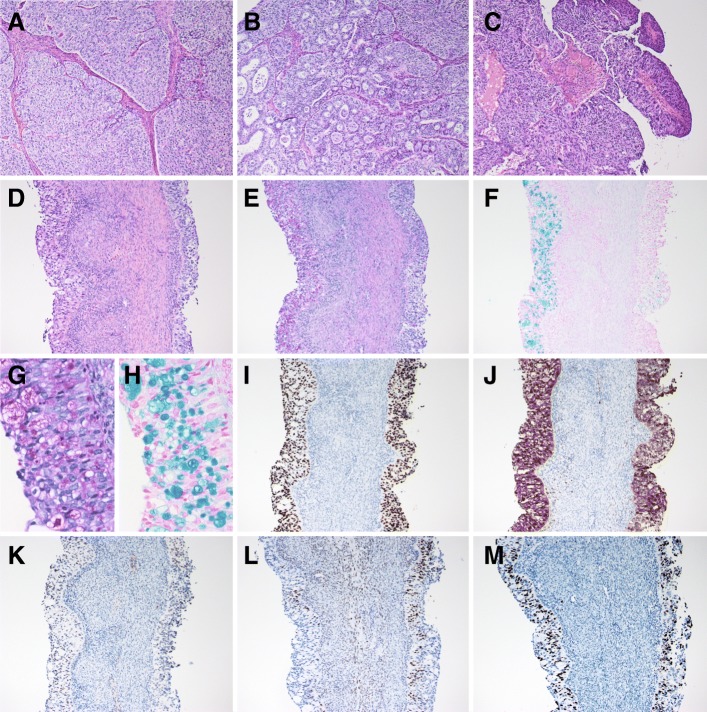
Pathological findings of the ovarian cancer. **a** − **d** the cancer cells show various morphology, including solid (**a**), pseudo-endometrioid (**b**) transitional-like (**c**) and mucin-producing (**d**) patterns. **e−h** Representative PAS (**e**, **g**) and Alcian blue (**f**, **h**) stained images of mucin-producing cancer cells. Note that ratio of mucin-producing tumor cells is different between the right and left side of septa. **i** − **m** Representative immunostained images of the p53 (**i**), p16 (**j**), WT1 (**k**), PgR (L) and Ki-67 (**m**)

The right ovarian tumor contained a serous fluid; the inside was multicystic and partially solid (Fig. 1b and c). Histologically, the cancer cells showed high-grade nuclear atypia and various histological patterns, including solid (Fig. 2a), pseudo-endometrioid (Fig. 2b), and transitional cell-like patterns (Fig. 2c). Such SET-type patterns were observed in approximately 90% of the tumor, while conventional HGSC histology was limited. In addition, Alcian blue and PAS staining demonstrated that some of the cancer cells contained intracytoplasmic mucin (Fig. 2d–h). The mucinous differentiated foci, which overlapped with other morphological patterns, were approximately 30% of the tumor, suggesting that the degree of deviation from the mucinous phenotype formed the heterogeneous multicystic image in this tumor (Fig. 1a). The cancer cells had spread into the left ovary and para-aortic lymph nodes, thus confirming the pathological FIGO stage IIIA (pT1bN1aM0). No benign and/or borderline mucin-producing epithelium, STIC, and endometriosis-related lesion were observed in the extensive histological analysis of the ovarian tumor (total 49 slides) and the fallopian tubes.

In our immunohistochemical analyses, the cancer cells showed diffuse positive staining for p53 (clone: DO-7; Fig. 2i); block positive for p16 (Fig. 2j); partial positive for WT1 (Fig. 2k), ER, PgR (Fig. 2l), CDX2 and PAX8; and negative for p40, p63, GATA3, Napsin A and vimentin (data not shown). The Ki-67 labeling index of the cancer cells was 60–80% (Fig. 2m).

**Fig. 3:**
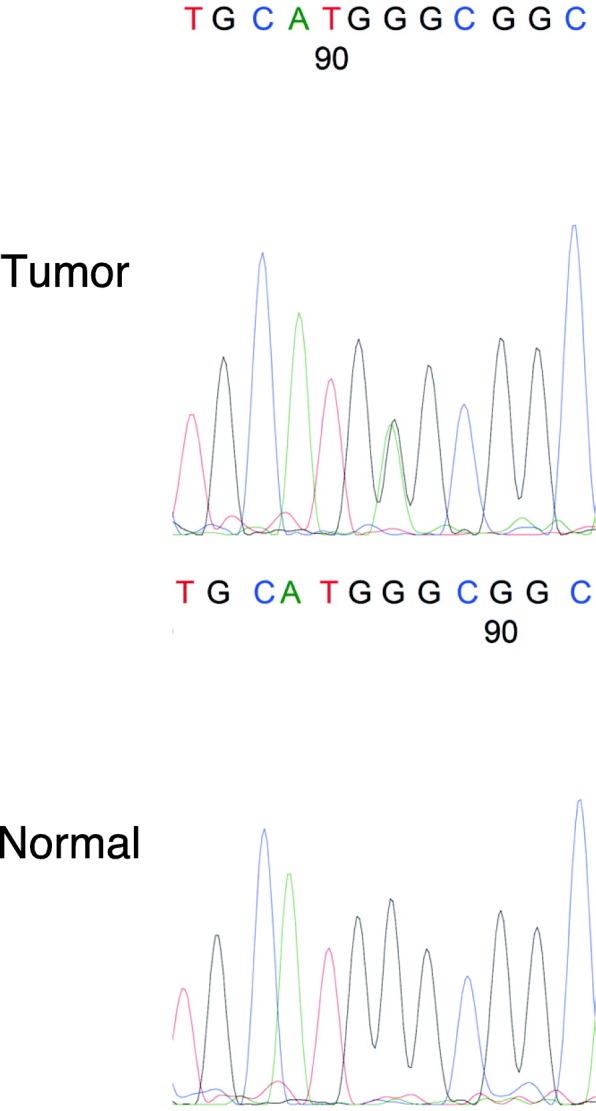
*TP53* mutation analysis of the ovarian tumor. A DNA sequence analysis of *TP53* exon 7 from the ovarian tumor and normal tissue with the reverse-primer. The sequence of the ovarian tumor shows biallelic pattern at the coding DNA reference number 730, indicating that HGSC harbors a missense mutation (p.G244S) in the *TP53* gene

Since an aberrant p53 expression pattern was displayed by immunohistochemistry, we performed *TP53* mutation analysis of ovarian cancer by direct sequencing according to the methods described previously [15]. The results showed that the cancer cells contain a c.730G>A mutation, which alters glycine to serine in codon 244 of exon 7 in *TP53* (Fig. 3). Finally, we diagnosed the ovarian tumor as a HGSC with SET feature and mucinous differentiation.

## Discussion

In the present case, the cancer cells showed high-grade nuclear atypia and histological heterogeneity, including SET-like and mucin-producing patterns. According to the first and second editions of WHO tumor classification [1, 16], mucin production may be found in serous tumors, particularly the serous borderline forms [16], but the mucins are almost entirely extracellular. In contrast, the mucin in the present case seemed to be intracellular and this mucin-producing phenotype mimicked that of a mucinous carcinoma or a minor ovarian epithelial cancer, seromucinous carcinoma [2]. Ovarian mucinous carcinoma contains mucus-scarce cells with marked nuclear pleomorphism [17] and occasionally harbors a *TP53* mutation [18, 19], whereas seromucinous carcinoma usually shows endometrioid carcinoma-like morphology and wild-type p53 immunophenotype [20]. In conclusion, these ovarian mucin-producing tumors should be ruled out in the present case.

On the other hand, the high-grade and/or various morphologies of the present case indicated type II carcinoma, such as SET-type HGSC. Consistent with the finding, the immunophenotype of the cancer cells resembled tubo-ovarian transitional cell carcinoma and HGSC [21, 22]. In addition, the relationship between *TP53* missense mutation and p53 overexpression is consistent with the recently published article about the correlation between *TP53* genotype and p53 immunophenotype in HGSC [23].

As described previously, transitional cell carcinoma is regarded as a variant of HGSC in the current WHO classification [2]. Interestingly, transitional cell carcinoma was defined as malignant transitional cell tumor without benign and/or borderline Brenner component [16, 24]. However, the majority of this category of ovarian cancers appeared with other histological types of malignant components, including serous, endometrioid, undifferentiated, or unclassified carcinoma [25]. Taking the molecular and morphological findings into consideration, it is reasonable to reclassify this entity of ovarian tumor as a HGSC SET variant. However, SET-type HGSC would be distinct from the conventional-type HGSC due to strong association with *BRCA* dysfunction that is a potent target of the poly-ADP ribose polymerase (PARP) inhibitor [14, 26–29].

In the present case, the major question was whether transitional cell carcinoma and/or SET-type HGSC show mucinous differentiation. Although the milestone papers of SET-type HGSC lacked the description of whether the SET feature includes the mucinous phenotype [14, 26], the AFIP atlas book illustrated the mucin secretion of malignant transitional cell tumor without a Brenner tumor component [30]. In addition, Silva et al. reported that 46 and 9 out of 88 TCC cases contained mucin-producing glands and small microcystic lesion, respectively [25]. Therefore, we believe that this ovarian tumor is a variant of HGSC because of the presence of pure high-grade carcinoma with SET-like morphology and *TP53* mutation and the absence of any ovarian benign and/or borderline tumor component, as revealed by the extensive pathological examination.

Besides the morphological pattern and molecular characteristics, the coexisting precursor and/or pre-malignant lesion is an important diagnostic clue for ovarian carcinoma. There are two candidate precursors of ovarian mucinous tumor: mature teratoma and Brenner tumor [31, 32]. In the present case, neither teratoma nor Brenner tumor element was found by radiological and pathological examination. Ovarian endometriosis, which is often associated with an ovarian seromucinous tumor, was also not detected [20]. Incidental lesion of uncertain significance, ovarian epithelial inclusion with mucinous differentiation [33] was also absent. Taken together, the results of our analyses suggested that no suspected premalignant lesions of the both mucinous and seromucinous tumors existed in the present case. In addition, absence of serous tubal intraepithelial carcinoma [34] was consistent with high-grade ovarian carcinoma, especially SET-type HGSC [26].

Surprisingly, mucin-producing phenotypes of HGSC were found in recent studies. Köbel and colleagues tried to establish a sophisticated immunohistochemical panel and algorithm for highly precise and reproducible classification of the five major ovarian carcinomas [3]. Although their attempt seemed to be practically successful, immunohistochemical and genetic findings of minor cases discorded with their morphological diagnosis. It is noteworthy that 2 of the 61 mucinous carcinomas revised the original histological type as HGSC following arbitration using combined biomarker-assisted review and next-generation sequencing. Their crossover of the histological type was similar to that in the present case, although such mucinous carcinoma was estimated to approximately 0.2% of the total ovarian epithelial carcinomas, according to their data. To clarify the clinicopathological significance of this extremely rare tumor, a case series study would be needed.

## Conclusion

We herein report the case of a HGSC with mucinous differentiation. This rare and unique tumor could be possibly misdiagnosed as a mucinous or seromucinous carcinoma especially when the diagnosis is based solely on morphological assessment of a small amount of histological sections. To avoid the misdiagnosis, extensive histological and immunohistochemical analyses should be performed when pathologists encounter high-grade mucin-producing ovarian carcinoma. In addition to the dedicated workup, we must recall that differential diagnosis of such tumors includes this rare variant of HGSC. In other word, mucinous phenotype in high-grade ovarian cancer cells is a candidate diagnostic clue of the HGSC SET variant. The present case shows that the unusual histological characteristic of high-grade serous carcinoma, the “SET” feature, could be extended to the solid, transitional, endometrioid and mucinous-like (STEM) feature. This comprehensive recognition of high-grade ovarian carcinoma, STEM, would contribute to the establishment of integrated histological and molecular-based tumor classification, which supports precise, reproducible and practical diagnosis and choosing an optimal personalized and/or molecular medicine in the future.

## Abbreviation

HGSC: High-grade serous carcinoma
LGSC: Low-grade serous carcinoma
SET: Solid, pseudo-endometrioid and transitional-like
FIGO: The International Federation of Gynecology and Obstetrics
WHO: World Health Organization
PARP: Poly-ADP ribose polymerase
STEM: Solid, transitional, endometrioid and mucinous-like
